# Mathematical modeling of apoptosis

**DOI:** 10.1186/1478-811X-11-44

**Published:** 2013-06-26

**Authors:** Kolja Schleich, Inna N Lavrik

**Affiliations:** 1 Division of Immunogenetics, German Cancer Research Center (DKFZ), Heidelberg, Germany; 2 Department of Translational Inflammation, Institute of Experimental Internal Medicine, Otto von Guericke University, Magdeburg, Germany

**Keywords:** Apoptosis, Death receptors, Systems biology, Modeling, Cancer

## Abstract

Apoptosis is a form of programmed cell death, which is fundamental to all multicellular organisms. Deregulation of apoptosis leads to a number of severe diseases including cancer. Apoptosis is initiated either by extrinsic signals *via* stimulation of receptors at the cellular surface or intrinsic signals, such as DNA damage or growth factor withdrawal. Apoptosis has been extensively studied using systems biology which substantially contributed to the understanding of this death signaling network. This review gives an overview of mathematical models of apoptosis and the potential of systems biology to contribute to the development of novel therapies for cancer or other apoptosis-related diseases.

## Introduction

**Figure 1:**
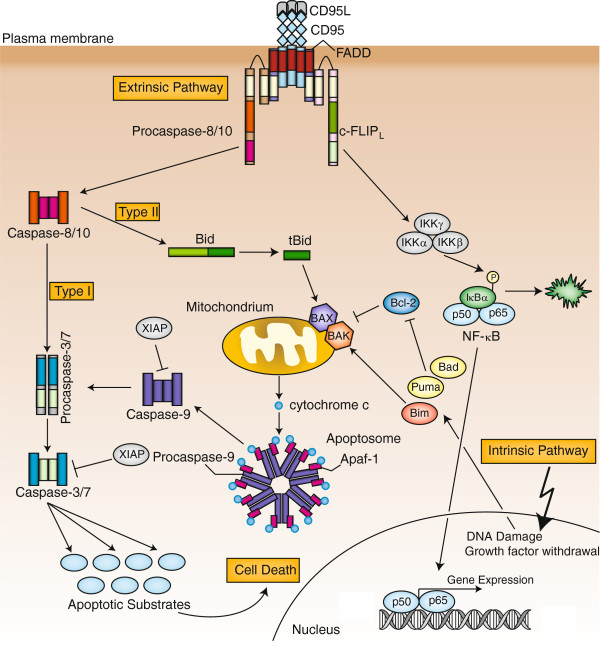
**Overview of apoptosis pathways**. Apoptosis can be induced by extracellular (extrinsic) or intracellular (intrinsic) stimuli. The extrinsic pathway is initiated *via* stimulation of DRs, *e*.*g*. CD95 or TRAIL-R, on the cell surface. Stimulation of CD95 results in the recruitment of different proteins including FADD, procaspase-8, procaspase-10 and c-FLIP proteins which form the death-inducing signaling complex (DISC). Procaspase-8 is activated at the DISC which is regulated by c-FLIP proteins. Active caspase-8 cleaves and activates effector caspase-3 and −7 and/or the Bcl-2 protein Bid. The truncated form of Bid (tBid) translocates to the mitochondria, triggering outer membrane permeabilization (MOMP) and the release of cytochrome c, as well as other pro-apoptotic proteins into the cytosol. Cytochrome c is involved in the formation of the apoptosome and procaspase-9 activation. Procaspase-9 also activates procaspase-3 and −7 resulting in massive caspase-3 activity and cell death. In CD95 signaling two different cell types are distinguished. Type I cells efficiently activate caspase-8 and subsequently effector caspases without requiring amplification through tBid-mediated MOMP. Type II cells, however, form less DISC and therefore rely on Bid cleavage and the intrinsic amplification loop. Additionally, CD95 can initiate non-apoptotic pathways, such as NF-κB or MAPK and cell survival. The intrinsic pathway is activated by various intracellular stimuli, such as DNA damage or growth factor withdrawal resulting in MOMP and subsequent effector caspase activation. Apoptosis is tightly controlled at the mitochondria by the Bcl-2 family of proteins.

Apoptosis is a certain form of programmed cell death (PCD) playing a key role in the development and homeostasis of multicellular organisms. Defects in the apoptotic pathway can lead to multiple diseases, such as neurodegenerative diseases, autoimmune diseases, AIDS and cancer [1]. In mammalian cells apoptosis can be induced by either extra- or intracellular stimuli, triggering the extrinsic or intrinsic pathway, respectively [2] (Figure 1). The extrinsic pathway is triggered by the stimulation of death receptors (DR) on the plasma membrane. DR stimulation leads to the formation of the death-inducing signaling complex (DISC) and initiator caspase-8 activation [3]. The intrinsic pathway can be triggered by chemotherapeutic drugs, irradiation or growth factor withdrawal and results in mitochondrial outer membrane permeabilization (MOMP), release of cytochrome C into the cytosol and procaspase-9 activation [4]. Both pathways eventually lead to the activation of effector caspases and the apoptotic phenotype including chromatin condensation, nuclear fragmentation, membrane blebbing, cell shrinkage and formation of apoptotic bodies [5-7]. Due to its extreme signaling outcome, *i*.*e*. life or death of the cell, apoptosis has to be tightly controlled at multiple levels and by various proteins. In order to better understand those signaling decisions numerous systems biology studies of apoptosis have been conducted. Systems biology is a relatively new field with the ultimate goal to understand biological processes in their entirety. Systems biology is based on the combination of experimental procedures with mathematical modeling that is supported by computer programs [8-10].

In this review we give an overview of systems biology approaches in apoptosis signaling with a focus on DR-induced apoptosis. Especially, we address a number of recent studies that provided new insights into apoptosis regulation since previous reviews have been written [11-14]. These studies include the determination of the DISC stoichiometry [15,16], molecular determinants of type I/type II apoptotic signaling [17], novel statistical methods of parameter estimation and model discrimination of cell death models [18], and using mathematical modeling of apoptosis for the development of novel anticancer therapies [19-21].

## Death receptor-induced apoptosis

DRs belong to the tumor necrosis factor receptor (TNF-R) family of proteins and initiate apoptosis upon stimulation with their respective ligands. All DRs are characterized by extracellular cysteine rich domains (CRD) as well as an intracellular approximately 80 amino acid long motif, the death domain (DD) [2,22]. CD95 (Fas/APO-1), TNFR1, TRAIL receptor 1 (TRAIL-R1) and TRAIL-R2 comprise the best characterized DRs [2,22,23]. Other DRs are DR3 and DR6, EDA-R and NGF-R [2,22,24]. The CD95- and TRAIL-R-mediated pathways of apoptosis are among the best studied signaling pathways and have been extensively used in systems biology studies. Binding of the natural CD95 ligand (CD95L) or agonistic antibodies, *e*.*g*. anti-APO-1 [25], to CD95 initiates the signaling cascade. A first event in DR signaling is the recruitment of the adapter protein Fas-associated death domain (FADD) to oligomerized receptors [3] (Figure 1). Subsequently, initiator caspases-8 and −10 and cellular FLICE-inhibitory proteins (c-FLIP) are recruited to the complex, that is named DISC [26,27] (Figure 1). Interactions between the molecules in the DISC are mediated by homotypic interactions. FADD is recruited *via* DD interactions with the receptor, caspase-8/10 and c-FLIP are recruited *via* death effector domain (DED) interactions with FADD [28]. Procaspase-8 contains two DEDs in its N-terminal part followed by a large (~20 kDa) and small (~10 kDa) catalytic subunit [26]. In the DISC procaspase-8 dimerizes, allowing the formation of an active site and activation of its catalytic activity [7,26,29-33]. The procaspase-8 dimer is further stabilized by cleavage between the large and small subunits [7,33]. Subsequent cleavage between the prodomain and the large subunit releases active caspase-8 into the cytosol and initiates the apoptotic cascade [26]. Caspase-8 activation is regulated by c-FLIP of which three isoforms are known: long (c-FLIP_L_), short (c-FLIP_S_) and Raji (c-FLIP_R_) [6,34-37]. They have structural similarities to procaspase-8. All three c-FLIP isoforms have a tandem DED at their N-terminus [38]. The long isoform, c-FLIP_L_, also has catalytically inactive caspase-like domains (p20, p12) [37,38]. Both short isoforms block procaspase-8 processing and activation at the DISC [6,27]. The long form, c-FLIP_L_, however, can accelerate procaspase-8 processing at low and intermediate concentrations, but inhibits it upon high concentrations [27,29,39]. Active caspase-8 further cleaves and activates the effector caspases-3 and −7 resulting in cell death [2] (Figure 1). In addition, it cleaves the pro-apoptotic Bcl-2 protein Bid which then translocates to the mitochondria triggering MOMP and amplification of the extrinsic signal [40,41]. In CD95 signaling two types of cells are distinguished [2] (Figure 1). Type I cells form high amounts of DISC allowing efficient procaspases-8 and −3/7 activation without requiring amplification through the cleavage of Bid [42]. Type II cells on the other hand activate caspase-8 at the DISC less efficiently and rely on the Bid-cleavage-mediated amplification loop [42]. Thus, type I cells still die when MOMP is inhibited, but type II cells do not. Apoptosis is further regulated by two other protein families. Inhibitors of Apoptosis proteins (IAP), with currently six human members: XIAP, c-IAP1, c-IAP2, NAIP, Bruce and survivin, can directly inhibit caspases [43]. The IAP family members share a conserved sequence motif, the baculoviral IAP repeat (BIR) domain, which is required for their function [7,44]. The family of Bcl-2 proteins has crucial functions in regulating MOMP with pro-apoptotic (*e*.*g*. Bax, Bak, Bid) and anti-apoptotic (*e*.*g*. Bcl-2, Bcl-X_L_) members [40,45]. Bcl-2 family members are characterized by the presence of the so-called Bcl-2 homology domain (BH1-BH4) [40,45].

Although best known as a ‘death receptor’, there is increasing evidence that CD95 also has functions in non-apoptotic signaling, such as tumor growth and invasion, proliferation and necroptosis [46-52]. It has been shown by numerous groups that CD95 activates NF-κB, a central mediator of inflammation, and MAPK signaling [46,53-59] (Figure 1).

## Mathematical formalisms used in systems biology

In systems biology experimental approaches are combined with mathematical modeling to understand complex behavior of cells and organisms. Experimental approaches and mathematical models are connected through a cyclic workflow [9]. Experimental data is used as input for mathematical models that, in turn, generate biological predictions. These predictions are then again verified by experimental approaches, thus completing the cycle. Experimental approaches, used in systems biology of apoptosis so far, include quantitative Western Blot, cell death assays, single cell analysis and mass spectrometry.

Numerous different modeling formalisms can be used to describe cellular systems and have been used, in particular, for modeling apoptosis. We shall briefly present the major modeling formalisms below.

In Boolean modeling protein-protein interactions are described qualitatively and kinetics of individual protein interactions are not taken into account. The signaling network is represented as a graph, signaling molecules are represented by the nodes and interactions by edges [60]. Each node can be either in an ‘off’ or ‘on’ state, represented by 0 and 1, respectively [60]. The state of each node depends on the state of its input nodes governed by logic rules (so-called *operators* or *gates*). Common boolean rules are the *AND*, *OR* and *NOT* operators. The *AND operator* will switch a node to the ‘on’ state only if all input nodes were in the ‘on’ state. On the contrary, an *OR operator* will switch a node to the ‘on’ state if one of the input nodes is in the ‘on’ state. The NOT operator is only used together with the other operators and is true if the corresponding input node is in the ‘off’ state [60]. Since each signaling component of the network can be only in two different states, boolean modeling cannot simulate temporal dynamics of protein concentrations.

In order to describe the quantitative temporal dynamics of a signaling network, ordinary differential equations (ODE) are used in systems biology [61,62]. ODE modeling assumes that the signaling molecules are highly abundant in the cell and well mixed. Therefore, stochastic effects and diffusion are neglected. In ODEs cellular reactions are usually described by mass-action kinetics [61-64] (see chemical textbooks for further general reading). A chemical reaction

$$A+B\overset{k_{1}}{\underset{k_{2}}{⇌}}C$$

can be transformed into the following system of coupled ODEs.

$$\frac{d\left[A\right]}{\mathrm{dt}}=\frac{d\left[B\right]}{\mathrm{dt}}=-k_{1}\cdot A\cdot B+k_{2}\cdot C$$

$$\frac{d\left[C\right]}{\mathrm{dt}}=k_{1}\cdot A\cdot B-k_{2}\cdot C$$

Such systems of ODEs can be solved numerically using standard computer software (*e*.*g*. Matlab). Usually dynamical pathway models include a large number of kinetic parameters which are not known and mostly cannot be measured experimentally. Therefore, parameters are estimated based on experimental data, *e*.*g*. kinetics of protein concentrations in the pathway of interest. Parameters can be estimated using the method of least squares [61]. Due to the high number of kinetic parameters compared to little experimental data, model fitting can yield multiple parameter values that can describe the data equally well. In order to test the robustness of a model sensitivity analysis is applied [61,63]. Furthermore, statistical methods can be used for parameter estimation of ODE models and are especially useful for discrimination between different model topologies with equally good fit to the same dataset [18]. ODEs assume homogenous distribution of interacting molecules and neglect spatial information. In order to take into account diffusion of molecules partial differential equations (PDEs) can be used [63].

Contrary to deterministic models, *e*.*g*. ODEs, stochastic models are based on the likelihood that two molecules interact. Another modeling approach is agent-based modeling [65-69]. This approach is a rules-based modeling approach in which each component of the model system is represented by an agent with a certain behavior based on its biological functions.

## Mathematical modeling of apoptosis

Mathematical modeling of apoptosis signaling reaches back more than a decade ago when Fussenegger *et al*. generated the first model of apoptosis [70]. This model was based on ODEs and described the apoptotic pathway both by extrinsic and intrinsic signals, but was not based on experimental data. The entirely theoretical model by Fussenegger *et al*. was followed by a number of ODE-based models supported by experimental data. The first model of this type described on the quantitative level the extrinsic apoptotic pathway, namely CD95 signaling [71]. This first experiment-based model of apoptosis initiated many more ODE-models of both the extrinsic and intrinsic pathways that appeared within the last decade. In addition, bistability of DR-induced apoptosis depending on initial procaspase-8 molecule numbers was demonstrated using an ODE model [72,73]. Besides ODE-based models other approaches have been applied to apoptosis modeling, including boolean modeling [74-77], Bayesian modeling [78], petri nets [79], cellular automata [80,81] and agent-based modeling [15].

Various mathematical formalisms applied to apoptotic signaling allowed to address a number of biological questions, *e*.*g*. how the switching between life and death in the cell occurs. In particular, it was possible to understand the contribution of different apoptosis regulators to apoptosis induction. For instance, considering extrinsic apoptosis signaling one might ask what the cell death stage-limiting steps are: DISC formation, activation of effector caspases, or cleavage of the caspase substrates. By combining quantitative Western Blot with a structured information ODE model our group revealed a threshold mechanism in CD95-initiated apoptosis which could also be verified experimentally [71,82]. In this work it was shown that activation of caspase-8 at the DISC is a stage-limiting step in extrinsic apoptosis in type I cells. Furthermore, it was shown that efficient DISC formation is the central control point of extrinsic apoptosis and the amount of the caspase-8 inhibitors c-FLIP in the cell is the major factor defining life/death decisions.

Similar questions, *e*.*g*. what the major regulators are and how the threshold is defined, were addressed using ODE modeling of intrinsic apoptosis. Models of the intrinsic apoptotic pathway demonstrated a crucial role of XIAP in caspase activation *via* the apoptosome [73,83]. Rehm *et al*. demonstrated that XIAP inhibits caspase activation only above a certain threshold, but enhances it at low concentrations [83]. Based on this model a simulation tool called APOPTO-CELL is available online [84].

The fascinating insights into the molecular mechanisms of apoptotic signaling were achieved by the combination of single cell analysis with mathematical modeling. For instance, spatio-temporal activation of caspases in TRAIL-induced apoptosis was understood in type II cells. It was shown that initiator caspases are active in the pre-MOMP phase while effector caspases are only active after MOMP [85]. In another model the same group analyzed cell-to-cell variability upon TRAIL stimulation [86]. They compared sister cells using time-lapse microscopy and FRET-based caspase-activity reporters and found that differences in signaling outcome are due to variations in protein levels.

Mathematical modeling of apoptosis also allowed determining and supporting quite some molecular paradigms. For a while the differences between type I and type II cells was an issue of debate. In a recent study Aldridge *et al*. studied the molecular determinants of type I vs. type II cells and demonstrated that the ratio of caspase-3 to XIAP is one of the major determinants of type I/type II decisions [17]. Another question that was unclear for a number of years is whether c-FLIP_L_ could indeed activate caspase-8 at the DISC. Only mathematical modeling was capable to predict the exact concentrations of the DISC components when c-FLIP_L_ could play an activating role [39]. These predictions were successfully confirmed by experimental data. Finally, a new mechanism of apoptosis signaling has been discovered recently using biochemical analysis, mass spectrometry and mathematical modeling. Namely, our group and the group of Marion MacFarlane determined the stoichiometry of the CD95 and TRAIL-R DISCs and found that caspase-8 outnumbers FADD at the DISC forming the so-called caspase-8 chains [15,16]. Using mathematical modeling we further analyzed the dynamics of the chain formation and found that the DISC is a very dynamic system and its stoichiometry, *i*.*e*. length of the caspase-8 chains, is defined by the strength of CD95 stimulation [15]. This finding adds another layer of complexity in caspase-8 activation and regulation of apoptosis in extrinsic signaling.

The non-apoptotic signaling features of CD95 have gained increasing interest, and, consequently, the concept of switching the phenotypes between apoptotic and non-apoptotic signaling also attracted the attention of systems biology research. To address the switch between apoptosis, necroptosis and survival Calzone *et al*. developed a Boolean model of TNF-R and CD95 signaling [77]. This was the first model of necroptosis that described the switch between necroptosis, apoptosis and survival as well as established the major molecules responsible for necroptosis induction. The other challenging question is how stimulation of DRs could also result in the induction of non-apoptotic pathways, such as NF-κB and MAPK pathways. Our ODE models of CD95-induced apoptosis found that DISC formation also leads to the induction of NF-κB and MAPK pathways [55,57]. Intriguingly, caspase-8 activity and the concentration of c-FLIP play an important role in NF-κB and MAPK induction [55,57]. Thus, our systems biology studies have demonstrated that the amounts of the main regulators of DR-induced apoptosis procaspase-8 and c-FLIP at the DISC also play a key role in the induction of non-apoptotic signaling.

After creating the basic models of the apoptosis pathways, currently systems biology of cell death is entering a next very important phase. The central question now is how defects in apoptotic networks are connected to various diseases and how apoptosis models could be used to develop better treatments. Especially important are opportunities that are provided by systematic consideration of the apoptotic network and possible connections to personalized medicine.

Apoptosis plays a central role in development and prevention of apoptosis is a hallmark of cancer. Despite extremely detailed understanding of the apoptotic pathway little progress has been made with respect to cancer therapy. Chemotherapy, radiotherapy and surgery remain the best treatment options so far. Systems biology studies of apoptosis may play an important role in the development of novel anticancer therapies. Indeed, recent studies from different groups used mathematical models of apoptosis and applied them to cancer cells [19-21]. This allowed finding out the major determinants of apoptosis resistance in cancer cells, which, in turn, provides a basis for the development of novel anti-cancer therapies targeting key components of the apoptotic pathway [19-21]. These first studies show ample opportunities that systems biology of the apoptotic network might provide with respect to cancer treatment and open new avenues for drug development.

## Conclusions

Systems biology of apoptosis in the last decade has undergone a tremendous development from the first theoretical model of apoptosis to models directly connecting defects in apoptosis networks to cancer. The next issues to address are the dynamics of apoptosis networks and cross-talk to other signaling pathways at the level of the cell, tissue and organism. A very ambitious question to address is what the role of the apoptotic network is in the diseases of the century *e*.*g*. ageing and metabolic diseases. Future challenges also involve further development of models of diseases associated to defects in apoptosis and successful development of anti-cancer therapies based on systems biology models.

## Competing interests

The authors declare there is no competing interest.

## Authors’ contribution

KS drafted the text, IL drafted the text. Both authors read and approved the final manuscript.
